# Supplementary material for: Allosteric regulation and crystallographic fragment screening of SARS-CoV-2 NSP15 endoribonuclease
Source: Nucleic Acids Res. 2023 Apr 28;51(10):5255–70. doi: 10.1093/nar/gkad314 (PMC10250223; doi:10.1093/nar/gkad314)
Supplement: gkad314_Supplemental_Files [file gkad314_supplemental_files.zip › Supplementary datasets.zip!/183/Refine_4/01-BUSTER/Cycle-5/shell.01/LogLik0.html]

```
# <Log-Likelihood> of null-hypothesis


<Log-Likelihood of null hypothesis>, 64732 all data           :      -1.27601

<Log-Likelihood of null hypothesis>,  3243 data in free set   :      -1.36234

<Log-Likelihood of null hypothesis>, 61489 data in working set:      -1.27146

```

```
```
